# Early stoma closure after low anterior resection is not recommended due to postoperative complications and asymptomatic anastomotic leakage

**DOI:** 10.1038/s41598-023-33697-9

**Published:** 2023-04-20

**Authors:** Ian Fukudome, Hiromichi Maeda, Ken Okamoto, Sachi Yamaguchi, Kazune Fujisawa, Mai Shiga, Ken Dabanaka, Michiya Kobayashi, Tsutomu Namikawa, Kazuhiro Hanazaki

**Affiliations:** 1 Department of Surgery, Kochi Medical School, Kohasu, Oko-Cho, Nankoku-City, Kochi 783-8505 Japan; 2 Cancer Treatment Center, Kochi Medical School, Kohasu, Oko-Cho, Nankoku-City, Kochi Japan; 3 Department of Surgery, Niyodo Hospital, Ino-cho, Agawa-Gun, Kochi, 1369 Japan

**Keywords:** Colorectal cancer, Gastroenterology

## Abstract

The safety of early stoma closure after lower anterior resection (LAR) for rectal cancer remains controversial. In this study, patients scheduled to undergo LAR and stoma creation for rectal cancer were recruited. In absence of anastomotic leakage on radiological examination, closure of the diverting ileostomy was performed within 2 weeks. The primary endpoint was incidence of the colorectal anastomosis leakage after early stoma closure. Because of the slow accrual rate, the study was closed before recruitment reached the planned number of patients (n = 20). Among the 13 patients enrolled between April 2019 and March 2021, early stoma closure was performed in seven patients (53.8%). Non-clinical anastomotic leakage, leakage identified only on radiological examination, occurred in five cases, resulting in rescheduling of stoma closure. One patient did not undergo early stoma closure due to ileus. After stoma closure, colorectal anastomotic leakage manifested in one case; its incidence rate was 14.2%. Surgical site infection occurred in 42.8% of patients. This study revealed that asymptomatic anastomotic leakage occurred frequently. Considering the low rate of successful cases and the high rate of complications, early stoma closure within 2 weeks after LAR should not be performed routinely. Trial registration: (UMIN000036382 registered on 03/04/2019).

## Introduction

Diverting ileostomy created at the time of lower anterior resection (LAR) of the rectum significantly reduces the possibility of anastomotic leakage and reoperation^1–4^. However, stoma affects the life of patients^5^ and hampers the continuation of postoperative adjuvant chemotherapy^6^. Thus, early stoma closure should be considered when anastomosis healing is confirmed. Nevertheless, the closure of the stoma is not performed early after the initial operation, according to conventional clinical practice. Indeed, a study demonstrated that ileostomy was closed at a median time of 4.1 months after LAR^7^.

Only a limited number of trials demonstrated the safety of early stoma closure (< 2 weeks)^8,9^. However, these studies were conducted with selected patients, and the evidence was insufficient to expand early stoma closure in routine clinical practice. Hence, this study focuses on evaluating the safety of early stoma closure within 2 weeks by recruiting patients in a prospective, single-center, and single-arm manner. The results would clarify if early stoma closure was feasible in patients, including the elderly, and answer if a larger clinical trial could be conducted to expand the strategy to routine clinical practice.

## Methods

### Patients

Patients with rectal cancer (Rs/Ra/Rb) who were scheduled for LAR and temporary ileostomy and voluntarily provided written consent after explanation of the study were included in this study. Patients requiring lateral pelvic lymph node dissection and preoperative chemoradiation were excluded from the study. Based on a previous study^8,9^, we prospectively recruited 20 patients to assess the safety of the procedure, and estimated that nearly half of the patients dropped before early stoma closure. Based on the volume of the surgery in the institution, 2 years of recruitment period was provided. The study protocol was reviewed and approved by the Institutional Review Board of Kochi Medical School (Approval number: 30-177) and was registered as UMIN000036382 on 03/04/2019.

### Endpoints

We hypothesized that the fear of manifestation of the non-clinical leakage (asymptomatic leakage proven only by radiological examination) was related to avoidance of early stoma closure in routine clinical practice. Thus, the primary endpoint to the incidence rate of postoperative colorectal anastomosis leakage, after early stoma closure (< 2 weeks), was allocated. The secondary endpoints included the rate of patients who received early stoma closure, rate of surgical site infection (SSI), and rate of postoperative complications, accuracy of diagnosis of anastomosis leakage, and length of hospitalization as a parameter of the medical cost.

### Procedure

LAR was performed by total mesorectal excision or tumor-specific mesorectal excision^10^ with regional lymphadenectomy. End-to-end anastomosis was performed using a double-stapling technique. When anastomosis was performed at the level of the levator ani muscle, a diverting ileostomy was created. In addition, the creation of a stoma was performed, according to the clinical judgment of the surgeons, with the consent of the patients. Water intake was started on the first postoperative day, and solid food intake was resumed on the second postoperative day. The existence of anastomotic leakage was assessed by contrast enema and plain computerized tomography (CT). When appropriate, early closure of stoma was performed (within 2 weeks of the initial surgery); otherwise, a conventional postoperative management was provided.

For stoma closure, functional end-to-end anastomosis with an automatic suturing device or layer-to-layer anastomosis by hand suturing was performed. The posterior layer of the fascia with the peritoneum and the anterior layer of the fascia were closed separately. After washing the subcutaneous tissue with normal saline, the skin was semi-closed circularly using an absorbable dermal suture. After stoma closure, solid food was resumed on the third postoperative day.

### Ethics

This study was approved by the Institutional Review Board for Clinical Research of the Kochi Medical School (IRB-107374) and was performed in accordance with the Declaration of Helsinki. The study was conducted after each patient provided written informed consent.

## Results

**Figure 1:**
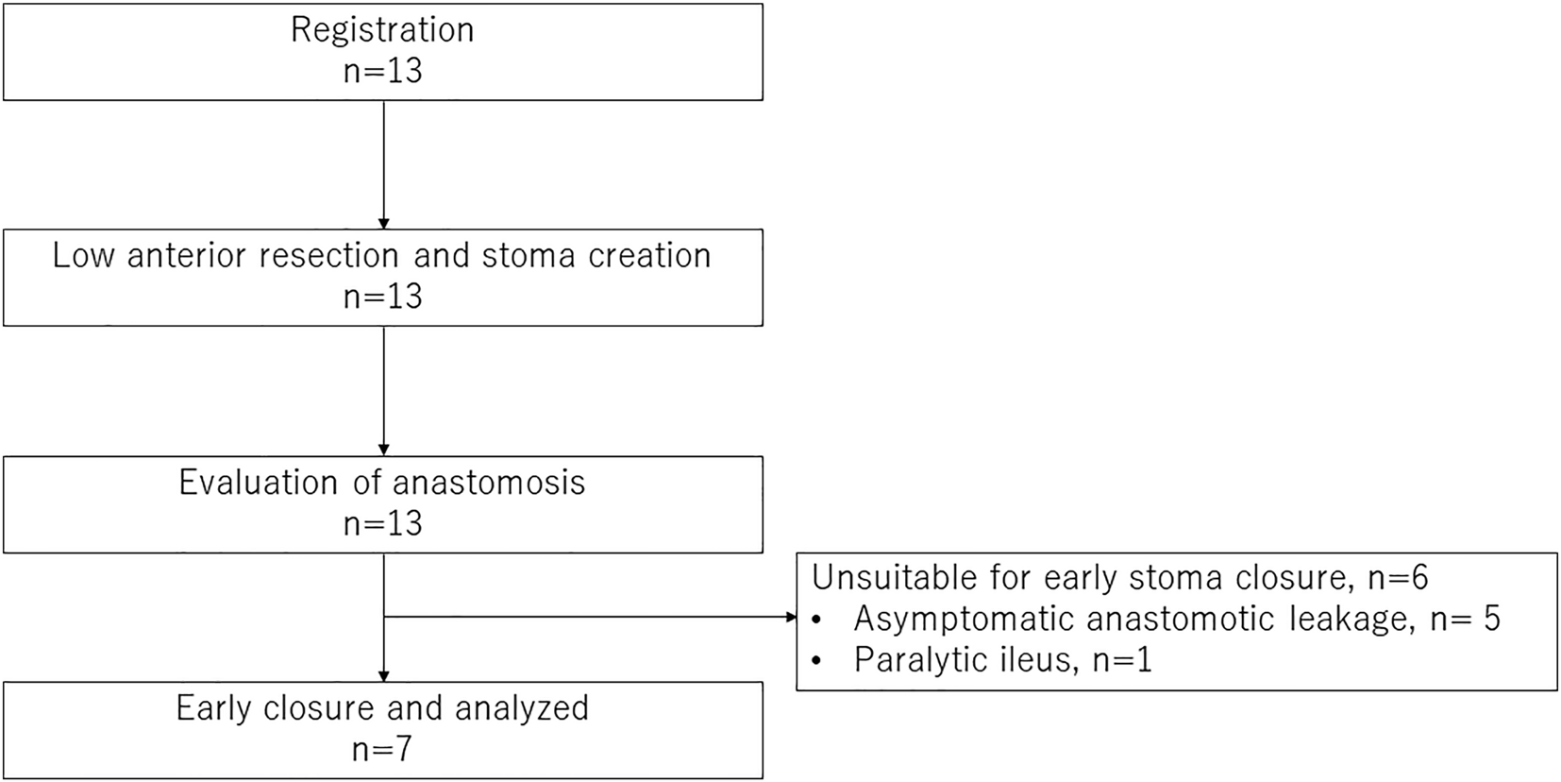
Recruitment and clinical course. Thirteen patients participated in this study. After planned surgery, all patients underwent an evaluation of colorectal anastomosis. Six patients did not receive early stoma closure due to suspected leakage (n = 5) or paralytic ileus (n = 1). Seven of the 13 patients underwent early stoma closure.

Due to the slow accrual rate, the study was closed after 13 patients were registered between April 2019 and March 2021 (Fig. 1). The Table 1 shows that the median age of the patients was 70 years (range: 37–84 years), the lower rectum was the most common, 38.4% had tumors larger than 40 mm, and one patient received preoperative chemotherapy. Laparoscopic and robotic surgeries were the most common surgical approaches, and open surgery was not chosen among the recruited patients. There were no obese patients (body mass index > 25 kg/m^2^) among the 13 patients.

**Table 1:** Clinical features of the patients.

Variables	Patients number, n = 13
Age	
Year, median [range]	70 [37–84]
Sex	
Male/Female	9/4
Tumor location	
Rs/Ra/Rb	1/6/6
Stage	
I	6
II	1
III	6
IV	0
Approach and surgery	
Lap-LAR	7
Robotic-LAR	6
Neoadjuvant chemotherapy	
Yes	1
Tumor size	
10–40 mm	8
> 40 mm	5

*Lap-* laparoscopic assisted, *LAR* lower anterior resection.

### Postoperative course of LAR

**Figure 2:**
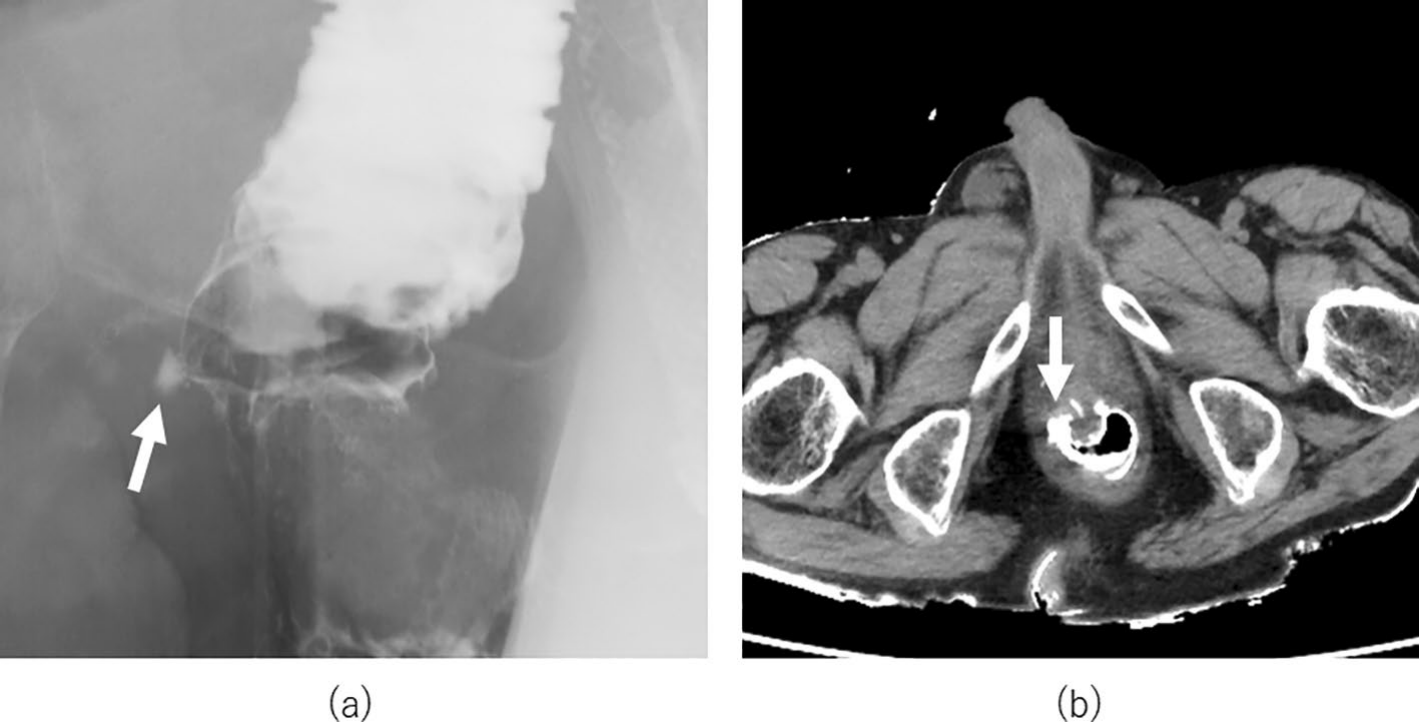
Contrast enema examination and computed tomography. A case of suspected anastomotic leakage is demonstrated. (**a**) The contrast medium flowed out of the intestinal tract. (**b**) The dehiscence of the anastomosis is difficult to identify on computed tomography.

All 13 patients underwent contrast enema and plain CT examinations (median: 8 days after LAR, range: 6–13 days); in five patients, anastomotic leakage was suspected (Fig. 2a and Fig. 2b). In one of the patients with suspected anastomotic leakage, fever occurred after examination and required further treatment by changing the drainage tube (Clavien–Dindo (CD) classification III complication, Table 2). Early stoma closure was postponed due to ileus in one case, which was treated conservatively. Therefore, stoma closure within 2 weeks was performed in seven patients (53.8%).

**Table 2:** Postoperative complications after LAR.

Complications	Patients number, n = 13
Total	5 (38.4)^a^
Paralytic ileus	1
Ileostomy-related ileus	1
Dehydration	1
Urinary retention	1
Fever after enema and CT examination	1

^a^The number in the parenthesis is percentage.

*CT* computerized tomography.

### Postoperative course after early and conventional stoma closure

Early stoma closure was performed approximately 12 days after LAR, ranging from 7 to 13 days (Table 3). The drainage tube used for LAR was often kept until a few days after stoma closure. In one patient, anastomotic leakage of colorectal anastomosis occurred one day post stoma closure, requiring emergency surgery and stoma creation again (CD classification IIIb complication). Intra-abdominal abscess (organ space SSI) due to a retrograde infection of the drainage tube occurred in 3 cases (42.8%), which was treated by drainage tube exchange (CD classification IIIa complication). The total length of hospital stays among patients undergoing early closed cases was 24 days, and the length of hospital stay after stoma closure was 16 days.

**Table 3:** Clinical course after early and conventional stoma closure.

	Early stoma closure, n = 7	Conventional stoma closure, n = 6
Interval between surgeries		
Day, median [range]	12 [7–13]	110 [55–219]
Complications^a^, n		
No complications	2	3
I	0	1
II	1	2
IIIa	3	0
IIIb	1	0
Total length of hospital stay^b^,		
Day, median [range]	24 [18–48]	27.5 [23–36]
Hospital stays after stoma closure		
Day, median [range]	16 [9–35]	9 [7–15]

^a^Complications are grouped according to the Clavien–Dindo classification.

^b^For patients undergoing conventional stoma closure, sum of the hospital stays at first and second admission was presented.

For patients who did not receive early stoma closure (n = 6), the stoma was closed approximately 110 days after LAR. Complications with III of the CD classification did not occur. The sum of the hospital stays of the first and second admission was 27.5 days and the hospital stay after stoma closure was 9 days.

## Discussion

In this study, only 53.8% of the registered patients (7 of 13 patients) were suitable for early stoma closure because asymptomatic (non-clinical) leakage was found in five cases on radiological examinations. In addition, one out of seven patients required emergency surgery due to peritonitis of colorectal anastomotic leakage. Early stoma closure may benefit limited number of patients. For instance, outlet obstruction of ileostomy could be resolved with an early stoma closure if the evaluation of the anastomosis indicates safety. However, disadvantage overweighs the potential advantage in general patients. After the present study, the plan for further study was discarded in this institute.

Anastomotic leakage after LAR is a serious complication, with an occurrence rate of 3–28%^11,12^, which often results in an emergency surgery. Thus, diverting ileostomy is created at the end of LAR because it reduces the rate of symptomatic anastomotic leakage and the possibility of reoperation^1–4^. None of the patients in this study experienced symptomatic anastomotic leakage. However, a high incidence of non-clinical (asymptomatic) anastomotic leakage was found. In addition, enrolled patients did not have any risk factors for colorectal anastomotic leakage, such as severe diabetes, high body mass index, simultaneous resection of other organs, perioperative transfusion, or a bulky tumor with obstruction^13–17^.

Two large clinical trials were conducted to evaluate early stoma closure^8,9^ at the time of this study. The focus was on the inclusion and exclusion processes rather than the conclusion that early stoma closure was conducted safely in selected patients. A study by Alves et al. assessed 260 patients after rectal surgery^8^. The author reported that 24% (n = 63) of the enrolled patients were excluded before randomization of the patients for early and conventional stoma closure. Of the patients, 7.9% (n = 20) had anastomotic leakage; while 16.9% (n = 43) of the patients had medical problems, including signs of active infection or organ failure in the postoperative period. This is in contrast to the study results, because only one patient with paralytic ileus in this study rescheduled early stoma closure due to medical conditions other than leakage. A recent study by Danielsen et al. also reported the details of the inclusion and exclusion processes^9^. Among the 418 evaluated patients, as many as 291 patients were excluded before randomization. 8.8% of the patients (n = 37) had suspected anastomotic leakage, and 38.0% (n = 159) were excluded after clinical evaluation. Even if the undiagnosed anastomotic leakage did not exist in these two trials, a significant proportion of the patients were excluded before performing early stoma closure, to a similar degree as that found in the present study.

The results of relevant clinical trials were recently disclosed by a study group in Switzerland^18^. They investigated the clinical outcomes of early and late stoma closure in a prospective randomized clinical trial. After excluding patients with abdominopelvic complications, the patients were invited and randomized. One day (median) before stoma closure, the anastomosis was examined to determine if closure was feasible. The authors found that four out of 37 patients (11%) were not suitable for early stoma closure due to delayed healing of the anastomosis. In addition, three out of 37 patients had leakage of large-bowel (colorectal) anastomosis after stoma closure. The clinical trial was prematurely terminated due to safety concerns. Based on the results, an asymptomatic leakage of colorectal anastomosis is not as low as previously speculated.

The present study also found that intra-abdominal abscesses occurred at a high rate (42.8%). Due to the possibility of anastomotic leakage after early stoma closure, a drainage tube was placed after low anterior resection (first operation) and was kept in place, which resulted in retrograde infection of the drainage tube. Thus, in early stoma closure, an attempt to secure patient safety caused complications, which is another disadvantage of early stoma closure.

A limitation of this study is that it was conducted in a single center with a small number of participants between 2019 and 2021. Studies with a larger number of patients in high-volume centers may result in a different conclusion, and the advancement of surgical techniques with sophisticated devices would change the practice. In addition, the lack of an established method for evaluating anastomosis should be taken into consideration, because it could be related to the high incidence of suspected anastomotic leakage like in the present study.

In conclusion, the present study demonstrated that only 53.8% of registered patients were suitable for early stoma closure because of the high rate of asymptomatic anastomotic leakage. In addition, postoperative complications related to the drainage tube undermine the benefits of early stoma closure, even in suitable patients. We believe that early stoma closure within 2 weeks after LAR could not be the standard treatment strategy, and the plan for further study was discarded from our institute.

## Data Availability

The datasets used and/or analyzed during the current study are available from the corresponding author on reasonable request.
